# ﻿Nomenclature and systematics of two *Cocconeis* species (Bacillariophyta) from Lake Baikal: *Cocconeisbaicalensis* and *Cocconeisskvortzowii*

**DOI:** 10.3897/phytokeys.247.131353

**Published:** 2024-10-18

**Authors:** Rinat Gogorev, Maria Yurchak, Irina Sokolova, Anton Glushchenko, Maxim Kulikovskiy

**Affiliations:** 1 Komarov Botanical Institute RAS, 2 Prof. Popova St., Saint Petersburg, 197022, Russia Komarov Botanical Institute RAS Saint Petersburg Russia; 2 К.А. Timiryazev Institute of Plant Physiology RAS, IPP RAS, 35 Botanicheskaya St., Moscow, 127276, Russia К.А. Timiryazev Institute of Plant Physiology RAS, IPP RAS Moscow Russia

**Keywords:** Baikal, *Cocconeis*, diatom, epitype, lectotypification, nomenclatural history, taxonomy

## Abstract

The study provides nomenclatural history, morphological characteristics and taxonomy of *Cocconeisbaicalensis* and *C.skvortzowii* described by B.V. Skvortzov from Lake Baikal. The lecto- and epitypification of the names are made, based on the K.I. Meyer and A.P. Skabichevsky materials, ensuring compliance with current nomenclatural standards. The morphological traits of these species were thoroughly examined using light microscopy (LM) nd scanning electron microscopy (SEM), leading to refined diagnoses and the clarification of previously confused synonymy.

## ﻿Introduction

Lake Baikal is the world’s deepest lake and diatoms are an integral part of its recent ecosystem. Monoraphid diatoms, including the genus *Cocconeis* Ehrenb., are poorly studied in Lake Baikal (Kulikovskiy et al. 2012, 2016a). Nine taxa of the genus *Cocconeis*, including 3 endemic ones, C.placentulavar.baicalensis, C.placentulavar.baikalensis and *C.nanoburyatica*, are known for the lake (Skvortzow and Meyer 1928; Skvortzow 1937; Gololobova 2012; Kulikovskiy et al. 2016a). Nomenclatural and taxonomic history of two of them, C.placentulavar.baicalensis and C.placentulavar.baikalensis, described by B.V. Skvortsov and currently reduced to synonyms for *C.skvortzowii* Sheshukova-Poretskaya, is confusing. The “disappearance” of one of the names from the taxonomic content of the genus, as well as different spelling of epithets, as Gololobova (2012) already had pointed out, and the citation of their authors in AlgaeBase (Guiry and Guiry 2024), made us feel the need to understand this issue.

This paper aims to typify the names of two *Cocconeis* taxa and to study their taxonomy and morphology based on typical specimens. All available published data were analyzed to outline their nomenclatural history. Based on original drawings, LM and SEM illustrations, lectotypes and epitypes are designated, as well as emended diagnoses are presented. Since the text of the new International Code of Nomenclature for algae, fungi, and plants (ICN) adopted by the XX International Botanical Congress, Madrid, Spain, July 2024, is not yet available, necessary references are given to the Shenzhen Code (Turland et al. 2018).

### ﻿Nomenclatural history

The variety CocconeisplacentulaEhrenbergvar.baicalensis B.V. Skvortzov et K.I. Meyer was described based on the materials of Meyer from Baikal Lake (Skvortzow and Meyer 1928). Since the authors of the name were not cited in the protologue, then, according to the *ICN*, its authorship is to be ascribed to the authors of the publication (Turland et al. 2018, Art. 46.3, Note 5), i.e. to both Skvortzov and Meyer. Nevertheless, Meyer in a subsequent study, cited the authorship of this name “CocconeisplacentulaEhr.var.baicalensis Skvortz.” (Meyer 1930, 341), and reported that the treatment of the Baikal diatoms collected by Meyer was made by Skvortzov alone (Meyer 1930, 183, 327). Later on, Skabichevsky (Skabichevsky 1974) on the basis of these notes by Meyer (1930) as well as the Meyer’s personal communication, concluded that Skvortzov should be cited as the only author of the diatom names published in Skvortzow and Meyer (1928). This view was adopted by Kociolek and Stoermer (1988, 96) and Williams and Reid (2001, 94). However, if we follow the laws of nomenclature, “In determining the correct author citation, only internal evidence in the publication... where the name was validly published is to be accepted” (Turland et al. 2018, Art. 46.8). On this ground, we consider both Skvortzov and Meyer as the authors of C.placentulavar.baicalensis (Skvortzow and Meyer 1928).

Later, Skvortzov (1946) published a new combination at the rank of species with a full and direct reference to the basionym (Skvortzow and Meyer 1928), but with a changed letter in the epithet: “*Cocconeisbaikalensis* (Skv. et Mey.) Skv.” (Skvortzov 1946, 19), which subsequently contributed to further confusion (see below). The species description was greatly changed and emended, especially regarding the density of striae, the range of which was increased and did not include data from the protologue, and new figures were also provided. We assume an erroneous measurement in Skvortzow and Meyer (1928) and we are clearly sure that these epithets refer to the same taxon (see below).

In 1937, Skvortzov described another variety from Baikal under almost the same name as in Skvortzow and Meyer (1928), Cocconeisplacentulavar.baikalensis Skvortzov, based on other material and with different from C.placentulavar.baicalensis illustration and description in protologue (Skvortzow 1937). Giving homonymic names to two different taxa was not the only case in Skvortzov’s scientific activity. In total, there are 58 «double» and even «triple» names in his 57 publications (M.A. Gololobova, pers. comm.). The epithets of the discussed varieties, as differing in one letter, are not full homonyms, but are so similar that they are likely to be confused (Turland et al. 2018, Art. 53.2, 53.3, Ex. 11). By analogy with Ex. 11, the epithets *baicalensis* and *baikalensis* should be treated as homonymous, with the later name being illegitimate.

To resolve the homonymy, a replacement name instead of the later homonym was published, cited as “*Cocconeisplacentula* Ehr. ... var. *Skvortzowii* (Skv.) Skabitsch.” (Sheshukova 1950, 85–86), with an incorrect statement of the status of the name, since parenthetical author citation suggests a name with a basionym (see Turland et al. 2018, Art. 49.1 and Art. 49.1, Note 1). The authorship of the combination was ascribed to Skabichevsky, which differs from authorship of the publication. The only reference to the replaced synonym is provided by the mention to “C.placentulavar.baicalensis Skv.”, without any reference to publication (Sheshukova 1950, 86). Since there is no explicit statement of Skabichevsky’s contribution to the publication (see the discussion below), as provided by Art. 46.2 (Turland et al. 2018), the authorship of the name should be ascribed to Sheshukova, the author of the Chapter “Subordo Monoraphineae” in Sheshukova (1950), and cited as “Skabitsch. ex Sheshukova” or simply “Sheshukova” (Turland et al. 2018, Art. 46.5, Ex. 31). An indirect reference to a replaced synonym is sufficient for valid publication of a replacement name before 1 January 1953 (Turland et al. 2018, Art. 41.3). Despite the spelling of the replaced epithet with a “*c*”, the description and the images of the variety clearly indicate to C.placentulavar.baikalensis Skvortzov (Skvortzow 1937). It remains unclear why the other variety, C.placentulavar.baicalensis B.V. Skvortzov et K. I. Meyer 1928, was not mentioned in Sheshukova (1950). According to Skabichevsky (1977), this was due to a vague description and a very schematic drawing in the protologue provided by Skvortzow and Meyer (1928, 11; Pl. 1, fig. 25).

Sheshukova (1951, 193) considered morphological peculiarities of C.placentulavar.skvortzowii essential enough to raise it to the rank of species, and published the new combination: *Cocconeisskvortzowii* “(Skv.) Sheshukova” (ascribing the authorship of the basionym to Skvortzov) with references to C.placentulavar.baicalensis “Skv.” and C.placentulavar.skvortzowii “(Skv.) Skabitsch.” (Sheshukova 1951, 193). Again, any mention of C.placentulavar.baicalensis B.V. Skvortzov et K.I. Meyer 1928 was missing there.

Skabitschewsky (1952), who most probably had overlooked both Sheshukova’s (1950, 1951) publications, also published a replacement name for C.placentulavar.baikalensis Skvortzov 1937, namely “Cocconeisplacentulavar.sibirica” (Skabitschewsky 1952). Due to the existence of an earlier replacement name (Sheshukova 1950) the Skabichevsky’s combination is nomenclaturally superfluous (Turland et al. 2018, Art. 52.1).

In his subsequent work, Skabichevsky (1977) raised the rank of both varieties of *C.placentula* to subspecific, and published corresponding combinations, with C.placentulasubsp.sibirica Skabichevskij being legitimate if treated as a replacement name rather than combination.

No specimens or localities are cited in the protologue of Cocconeisplacentulavar.baicalensis B.V. Skvortzov et K.I. Meyer (Skvortzow and Meyer 1928, 11) [≡*Cocconeisbaicalensis* (B.V. Skvortzov et K.I. Meyer) Skvortzov]. In the publication (Skvortzow and Meyer 1928), there is only the list of 36 gatherings, but without exact places of finding of particular taxa (Skvortzow and Meyer 1928, 2). The names of taxa were associated with collection sites by Meyer, who listed 8 localities of C.placentulavar.baicalensis (Meyer 1930, 341) corresponding to nine gatherings (Skvortzow and Meyer 1928, 2). Opposite, material of Skvortzow (1937), including C.placentulavar.baikalensis Skv., was obtained from a little bottom sample collected by Prof. K.I. Meyer at the depth of 33 meters near the Olhon Gate of Baikal Lake July 29, 1916 (Williams and Reid 2001, 297).

Such difficult nomenclature history of Skvortzov’s taxa of the genus *Cocconeis* led to mistakes, including in databases that were popular among diatomologists such as Algabase and DiatomBase. That was considered and described in Yurchak et al. (2023).

The nomenclatural history outlined above clearly demonstrates that there are 8 validly published names, both legitimate and illegitimate, referring to 2 independently described taxa (originally in the rank of varieties) and, accordingly, 2 groups of homotypic synonyms.

Thus, based on the significantly different valve morphology, we accept two independent *Cocconeis* species in the genus from Baikal, the basionyms of which are homonyms, but have different years of description and types.

## ﻿Materials and methods

In the work, some materials of K.I. Meyer and A.P. Skabichevsky were investigated, including those described in the protologues of the *Cocconeisbaicalensis* and *C.skvortzowii* (Table 1).

**Table 1.** List of specimens and slides.

Slide/Specimen	Data of sampling	Location in Baikal	*Legit*	Original label	Deposition, slide number
Specimen authenticum	n.d.	n.d.	K.I. Meyer	Baikal. 9.8. zavar	Diatom collection LE
Specimen authenticum	26 May 1921	n.d.	K.I. Meyer	N 96. Vemetlenskiy zal., 26/V 21	Diatom collection LE
Specimen authenticum	n.d.	n.d.	K.I. Meyer	N 2. Baikal 25.	Diatom collection LE
Specimen authenticum	11 June 1925	West Baikal, railway station Marituy	K.I. Meyer	N 5. Baikal 25 g. Maritui	Diatom collection LE A0002317
Specimen authenticum	n.d.	n.d.	K.I. Meyer	N 9. Baikal 25	Diatom collection LE
Specimen authenticum	n.d.	Marituy	K.I. Meyer	N 10. Baikal 25, Maritui	Diatom collection LE
Specimen authenticum	n.d.	Marituy	K.I. Meyer	N 13. Baikal 25 g. Maritui	Diatom collection LE
Specimen authenticum	n.d.	Cape Polovinny, near Marituy	K.I. Meyer	N 18. Baikal 25 g. B. “Polovinni”	Diatom collection LE
Specimen authenticum	n.d.	Cape Polovinny, near Marituy	K.I. Meyer	N 19. Baikal 25 g. B. “Polovinni”	Diatom collection LE
Specimen authenticum	n.d.	Proval Bay, Village Dubinino	K.I. Meyer	N 38. Baikal 25, Proval u m. Prorzy	Diatom collection LE
Specimen authenticum	n.d.	River Selenga	K.I. Meyer	N 53. Baikal 25, r. Selenga	Diatom collection LE A0004246
Specimen authenticum	n.d.	River Selenga	K.I. Meyer	N 61. Baikal 25, Selenga	Diatom collection LE A0004247
Specimen authenticum	n.d.	River Selenga	K.I. Meyer	N 69. Baikal 25 g. r. Selenga	Diatom collection LE A0004248
Specimen authenticum	n.d.	River Selenga	K.I. Meyer	N 70. Baikal 25 g. Selenga	Diatom collection LE A0004249
Specimen authenticum	n.d.	River Selenga	K.I. Meyer	N 71. Baikal 25 g. Selenga	Diatom collection LE A0004250
Specimen authenticum	n.d.	Left from River Angara	K.I. Meyer	N 88. Baikal 25 g. M. Tolsty	Diatom collection LE
Specimen authenticum	14 July 1925	Village Oymur	K.I. Meyer	N 125. Baikal 25 g. laguna Oimur, 14-VII-25	Diatom collection LE
Specimen authenticum	n.d.	n.d.	K.I. Meyer	N 154. Baikal 25, Istyakskiy sor	Diatom collection LE
Specimen authenticum	19 July 1925	n.d.	K.I. Meyer	N 159. Baikal 25, Proval, 19-VII-25	Diatom collection LE
Specimen authenticum	n.d.	City Babushkin	K.I. Meyer	N 216. Baikal 25, Mysovka	Diatom collection LE
Specimen authenticum	4 August 1925	n.d.	K.I. Meyer	N 232. Baikal 25, Istyakskiy sor, 4-VIII-25	Diatom collection LE
Specimen authenticum	n.d.	Cape Kotelnikovskiy	K.I. Meyer	N 302. Baikal 26 g. Koteln. mys, Khimeinovy ist.	Diatom collection LE A0004251
Specimen authenticum	27 June 1926	Cape Kotelnikovskiy	K.I. Meyer	N 303. Baikal, “Kotelnikovi” istochnik, 27/VI 26	Diatom collection LE A0004252
Specimen authenticum	30 June 1926	Cape Kotelnikovskiy	K.I. Meyer	N 318, “Kotelnikovi Maiak”, *Aegagrophila*, 30/VI-26	Diatom collection LE, A0004242, A0004243
Specimen authenticum	n.d.	Boguchanskaya Gulf	K.I. Meyer	N 336. Baikal 26, “Bogutchanskaia” guba	Diatom collection LE, A0004244, A0004245
Specimen authenticum	n.d.	Between Khargino and Buguldeyka	K.I. Meyer	N 524. Baikal, m. Mar, m. Krasny [Yar]	Diatom collection LE
Specimen authenticum	2 June 1927	Village Onguren	K.I. Meyer	N 632. Baikal, Onguren, 2-VI-27	Diatom collection LE
Specimen authenticum	20 June 1928	Between Murino and Vydrino	K.I. Meyer	N 654. g. Tan’, 20/VI 28	Diatom collection LE
Specimen authenticum	21 June 1928	n.d.	K.I. Meyer	N 661. Mezhdu Utulikom i Teler, 21/VI 28	Diatom collection LE
Specimen authenticum	29 June 1928	Opposite Island Olkhon	K.I. Meyer	N 695-96. o. Listvennichny, 29/VI 28	Diatom collection LE
Specimen authenticum	4 July 1928	Cape Buchenkova	K.I. Meyer	Baikal, g. Buchenkova, 4-VII-28	Diatom collection LE
Specimen authenticum	11 July 1928	Island Bolshoy Ushkaniy	K.I. Meyer	N 751. Baikal, B. Ushkaniy o., 11-VII-28	Diatom collection LE
Specimen authenticum	19 July 1928	Nord-East Baikal, near Mountain Turkukit	K.I. Meyer	N 820. Baikal, u r. Shengangda, s gl. 8 m, 19-VII-28	Diatom collection LE
Specimen authenticum	31 July 1928	n.d.	K.I. Meyer	N 904. Baikal, t. m. Kosy, 31-VII-28	Diatom collection LE
Specimen authenticum	n.d.	Nord-West Baikal, Cape Elokhin	K.I. Meyer	N 909. Baikal 28, m. Elokhin	Diatom collection LE A0002318
Specimen authenticum	n.d.	n.d.	K.I. Meyer	Radzimovski	Diatom collection LE
Slide authenticum	23 June 1925	East Baikal, Cape Ostrovki	K.I. Meyer	N 6 (= 7, = 53 [under label]), r. Selenga, Ostrovki, 23 VI 1925	Diatom collection LE A0002284
Slide authenticum	26 June 1925	Selenga River delta, channel Motumga	K.I. Meyer	N 4 (= 4, = B?), r. Selenga, prot. Motumga, 26 VI 1925	Diatom collection LE A0002285
Slide authenticum	30 June 1925	Selenga River delta, near Village Merkutov	K.I. Meyer	N 1 (= 2), r. Selenga, Merkushevo, 30 VI 1925	Diatom collection LE A0002286
Slide authenticum	30 June 1925	Village Merkutov	K.I. Meyer	N 2 (= 7c), r. Selenga, Merkusheva, 30 VI 1925	Diatom collection LE A0002287
Slide authenticum	30 June 1925	Village Merkutov	K.I. Meyer	N 3 (= 7?), r. Selenga, Merkushevo, 30 VI 1925	Diatom collection LE A0002288
Slide authenticum	1 July 1925	Village Merkutov	K.I. Meyer	N 5 (= 73), r. Selenga, d. Merkushevo, 1 VII 1925	Diatom collection LE A0002289
Slide authenticum	n.d.	Village Kultuk	V.P. Sukachev	Oz. Baikal, u Kultuk‘, *Chara*	Diatom collection LE A0002290
Slide	20 July 1965	Island Bolshoy Ushkaniy	A.P. Skabichevsky	SZCZ BL18566, SZCZ BL18567, SZCZ BL18568, SZCZ BL18569, SZCZ BL18570, SZCZ BL18571, SZCZ BL18572, SZCZ BL18573, SZCZ BL18574, SZCZ BL18575, SZCZ BL18676	Collection of Maxim Kulikovskiy
Specimen	20 July 1965	Island Bolshoy Ushkaniy, sand, 42 m depth	A.P. Skabichevsky	SZCZ BL15645	Diatom collection SZCZ
Specimen	20 July 1965	Island Bolshoy Ushkaniy, epiphithic assemblage on macroalgae thallus, 8 m depth	A.P. Skabichevsky	SZCZ BL15646	Diatom collection SZCZ
Specimen	20 July 1965	Island Bolshoy Ushkaniy, epilithon assemblage on macroalgae thallus, 4 m depth	A.P. Skabichevsky	SZCZ BL15647	Diatom collection SZCZ
Specimen	20 July 1965	Island Bolshoy Ushkaniy, sand, 42 m depth	A.P. Skabichevsky	SZCZ BL15650	Diatom collection SZCZ
Specimen	20 July 1965	Island Bolshoy Ushkaniy, sand, south shore, 14 m depth	A.P. Skabichevsky	SZCZ BL15657	Diatom collection SZCZ

Sample preparation for light and scanning electron microscopy included the dissolution of samples with diatoms in concentrated hydrogen peroxide. The samples were treated with 10% hydrochloric acid to remove carbonates and were then rinsed several times with deionized water every 12 hours. Afterwards the samples were boiled in concentrated hydrogen peroxide (~37%) to mineralize the organic matter. They were washed again with deionized water four times at 12-hour intervals. After decantation and filling with deionized water up 25 to 100 ml, the suspension has been spread onto cover slips and left to dry at room temperature.

Permanent diatom preparations were mounted in Naphrax^®^. LM observations were performed by means of a Nikon Eclipse E600 equipped with a Plan-apochromatic oil immersion objective (×100/n.a. 1.4) and Nikon DS-5M digital camera, a Zeiss AxioScope A1 microscope equipped with a Plan-apochromatic oil immersion objective (×100/n.a. 1.4, DIC) and a Zeiss Axio Imager А2 equipped with a EC Plan-Neofluar oil immersion objective (×100/1.30, DIC) and Axiocam 506 color digital camera. Valve ultrastructure was examined using a Hitachi S4500 and JSM-35С field emission scanning electron microscopes with an accelerate voltage 10–35 kV. The LM Zeiss Axio Imager.А2 and SEM JSM-35С microscopes are an equipment of The Core Facilities Center “Cell and Molecular Technologies in Plant Science” at the Komarov Botanical Institute RAS (Saint Petersburg, Russia).

The specimens and slides are deposited in LE (Komarov Botanical Institute of RAS, Saint Petersburg, Russia), diatom collection SZCZ (University of Szczecin, Szczecin, Poland), and the collection of M.S. Kulikovskiy (Herbarium of the K.A. Timiryazev Institute of Plant Physiology of RAS, Moscow, Russia).

The measurements of length and width of valves, density of striae and areolae were carried out using the program ImageJ. We calibrated scale bars in the program according to the lines of pictures for correct measurements. We measured the length of 10 striae/areolae in the middle between (1) an axial area and a valve face border, (2) a center and apex of valve. Then we recounted their density in 10 µm by using arithmetical proportion. If there were not 10 striae/areolae on valve, we used a smaller number.

### ﻿Terminology

**LM** light microscopy.

**RV** raphe valve.

**RVVC** raphe valve valvocopula.

**SEM** scanning electron microscopy.

**SV (RLV)** sternum (rapheless) valve.

**Leg.***Legit* (latine), сollected.

The common terminology follows Romero and Rivera (1996) and Gogorev et al. (2018, 2024).

## ﻿Results

We propose here three terms that are needed to define and distinguish “unusual” morphological structures found in some taxa of the genus *Cocconeis* (contra mantle) or more widely represented in diatoms, but not found in the terminology used.

Contra mantle — the mantle (or its part) of the raphe valve in some *Cocconeis* species is not directed towards the adjacent rapheless valve, but in the opposite direction.

Ghost areolae – small depressions located in striae or irregularly and visible in LM as usual/normal areolae.

Ghost raphe — a rudimentary raphe on rapheless valve of some monoraphid diatoms filled in with silica during valve morphogenesis and distinguishable in mature valve: in SEM**GR** is externally presented as a small groove, less often as two grooves corresponding to two branches of the raphe; in LM**GR** often looks like a normal raphe, so a rapheless valve can be confused with a raphe valve.

**Figure 1.**
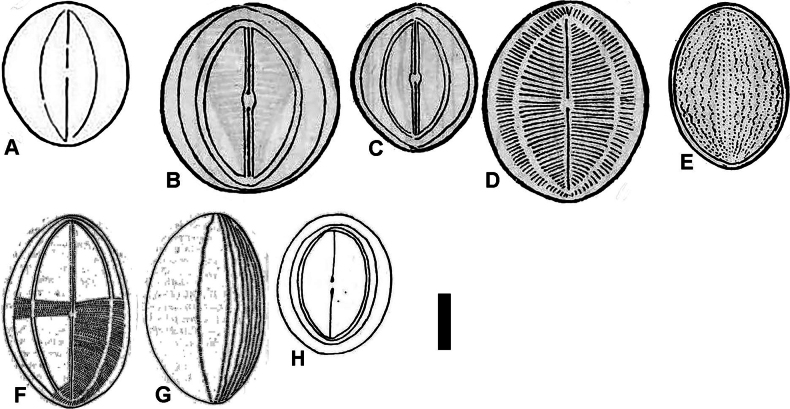
Reproduction of original drawings of *Cocconeisbaicalensis***A** protologue, Skvortzow and Meyer (1928), pl. 1, fig. 25 **B–E**Skvortzov (1946), table 5, figs 9–11, table 3, fig. 24 **F–H**Skabichevsky (1977), fig. 2, 6–8 **A–D, F, H** raphe valve **E, G** sternum valve. Scale bar: 10 µm.

**Figure 2.**
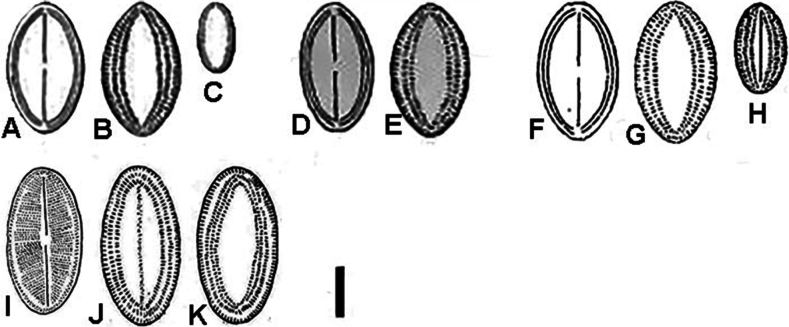
Reproduction of original drawings of *Cocconeisskvortzowii***A–C** protologue, Skvortzow (1937), pl. 5, figs 7, 8, 5 **D, E**Sheshukova (1950), 86, table 30, fig. 10а, b, reproduction from Skvortzow (1937)**F–H**Sheshukova (1951), 193, fig. 104, а–c **I–K**Skabichevsky (1977), 127, figs 2, 9, 11, 10 **A, D, F, I** raphe valve **B, C E G, H, J, K** sternum valve. Scale bar: 10 µm.

Below we provide complete taxonomical citations of two species with nomenclatural remarks and indications of mistakes and inaccuracies committed in the referred publications (Tables 2, 3, Figs 1, 2).

**Table 2.** Synonyms and traits of *Cocconeisbaicalensis* (≡ var.baicalensis Skvortzow et Meyer 1928) in published sources.

Epithet spelled exactly as in the source	Distribution source (specimens)	Valve measurements			Reference
		Length (μm)	Width (μm)	Striae in 10 μm	
CocconeisplacentulaEhrenb.var.baicalensis	Baikal (thematic monograph on Baikal), no data on distribution or samples	25.5	22.1	14 (RV)	Skvortzow and Meyer (1928, p. 11, Pl. 1, fig. 25)
CocconeisplacentulaEhr.var.baicalensis Skvortz.	Baikal expedition of the USSR Academy of Sciences, 1916, 1925–1929: r. Selenga, okolo Merkusheva (No. 15–16, Selenga River, near the village–Merkutov, 30 VI 1925), bukhta Peschanaya (No. 20, Pestchannaia [Peschanaya] Bay, from *Aegagrophilapulvinata*, 25 VII 1925), Kotel’nikovskiy mayak (No. 21, 27, “Kotelnikovi Maiak” [lighthouse], near shore, from *Ulothrix*, at depth 10–15 m, 30 VI 1926), p. Guyel’ga (No. 22, Guelga village, from *Ulothrix*, 2 VII 1926), Boguchanskaya guba (No. 23, 29–30, “Bogutchanskaia” gulf, near “Tonki” cape, at depth 5–7 m, 5 VII 1926), Maloye more (Kharansa Island, 1927), m. Elokhin, Barguzinskiy zal. (guba Buchenkova, 1928 g.) [Cape Elokhin, Barguzin Bay (Buchenkova Bay, 1928)]	25.1	22.1	14 (RV)	Meyer (1930, 341)
*Cocconeisbaikalensis* (Skv. et Mey) Skv.	Hab. Siberia, in lacum Baikal	14–30.6	14–23	22–24 (SV), 18–20 (RV)	Skvortzov (1946, 19, table 3, fig. 24; table 5, figs 9–11)
CocconeisplacentulaEhr.subsp.baicalensis (Skv.) Skabitsch.	Lake Baikal, eastern shore in the area of Davsha Bay and Cape Pongonye (Tolsty), from *Chaetomorphacurta*, depth 30–50 m. Material from the Expedition of the Limnological Institute, June 1965	10–23	10–19	25–27 (SV), 23–25 (RV)	Skabichevsky (1977, 127, fig. 2, 6–8)

**Table 3.** Synonyms and traits of *Cocconeisskvortzowii* (≡ var.baikalensis Skvortzow, 1937) in published sources.

Epithet spelled exactly as in the source	Distribution source (specimens)	Valve measurements			Comments	Reference
		Length (μm)	Width (μm)	Striae in 10 μm		
Cocconeisplacentula(Ehr.)var.baikalensis	p. 297: little bottom sample collected by Prof. K.I. Meyer at the depth of 33 meters near the Olhon Gate of Baikal Lake July 29, 1916	12–24	6.8–14	18 (SV), 30 (RV)	description and drawings of the taxon do not correspond to the “first” epithet	Skvortzow (1937, 310, Pl. 5, figs 5(?), 7, 8)
*Cocconeisplacentula* Ehr. var. *Skvortzowii* (Skv.) Skabitsch.	Baikal Lake	12–24	7–14	18 (SV), 30 (RV)	n.d.	Sheshukova (1950, 86, table 30, figs 10а, b)
*Cocconeisskvortzowii* (Skv.) Sheshukova	Baikal Lake	14–36	8–22	15–20 (SV)	emended description and all drawings correspond to protologue (Skvortzow 1937)	Sheshukova (1951, 193, fig. 104, а–c)
Cocconeisplacentulavar.sibirica	n.d.	n.d.	n.d.	n.d.	n.d.	Skabitschewsky (1951, 36)
CocconeisplacentulaEhr.subsp.sibirica (Skv.) Skabitsch.	Baikal Lake, eastern shore in the area of Davsha Bay and Cape Pongonye (Tolsty), from *Chaetomorphacurta*, depth 30–50 m. Material from the Expedition of the Limnological Institute, June 1965	11–32	7–15	13–15 (SV), 20 (RV)	Based on Skvortzov’s description in Skvortzow (1937), author incorrectly provided data on striae density of RV and SV (confused with each other)	Skabichevsky (1977, 127, figs 2, 9–11)

n.d. – no data.

### ﻿ Cocconeis baicalensis

Taxon classificationPlantaeCocconeidalesCocconeidaceae

(B.V. Skvortzov & K.I. Meyer) B.V. Skvortzov 1946 in Zapiski Kharbinskogo Obshchestva Estestvoispytatelei i Etnografov, 2. Botany: 19, pl. 3, fig. 24; pl. 5, figs 9–11 (as “ baikalensis” (Skv. et Mey.) Skv.) emend. Gogorev & Yurchak

5C97E54C-71D2-5430-B3F2-738B8940C257

Cocconeis placentula Ehrenberg var. baicalensis Skvortzov & K.I. Meyer 1928 in *Proceedings of the Sungaree River Biological Station* 1, 5: 11, pl. 1. fig. 25, earlier homonym of C.placentulavar.baikalensis Skvortzov 1937. Basionym.
≡ CocconeisplacentulaEhrenbergsubsp.baicalensis (Skvortzov & K.I. Meyer) Skabichevskij 1977 in *Prirodnye kompleksy nizshikh rastenii Zapadnoi Sibiri*: 127, fig. 2, 6–8 (with authorship of the basionym “Skv.”). Synonym.

#### Type materials.

***Lectotype*** • (designated here): Baikal, Cape Kotelnikovskiy, No. 318, 30 June 1926 [N 318, “Kotelnikovi Maiak”, *Aegagrophila*, 30/VI-26], leg. K.I. Meyer, permanent slide No. 318a, LE A0004242.

***Isolectotype*** • Baikal, Cape Kotelnikovskiy, No. 318, 30 June 1926 [N 318, “Kotelnikovi Maiak”, *Aegagrophila*, 30/VI-26], leg. K.I. Meyer, permanent slide No. 318b, LE A0004243.

***Epitype*** • (designated here): figures here represented by Fig. 9C (Baikal, specimen BL15645, leg. A.P. Skabichevsky, 20 July 1965).

#### Type locality.

Russia, Lake Baikal, Barguzin Bay, Bay Pestchannaya, Boguchanskaya Gulf, Cape Elokhin, Cape Kotelnikovskiy, Island Bolshoy Ushkaniy, Maloe More, Marituy, Selenga River, Village Guelga.

#### Description.

**Figure 3.**
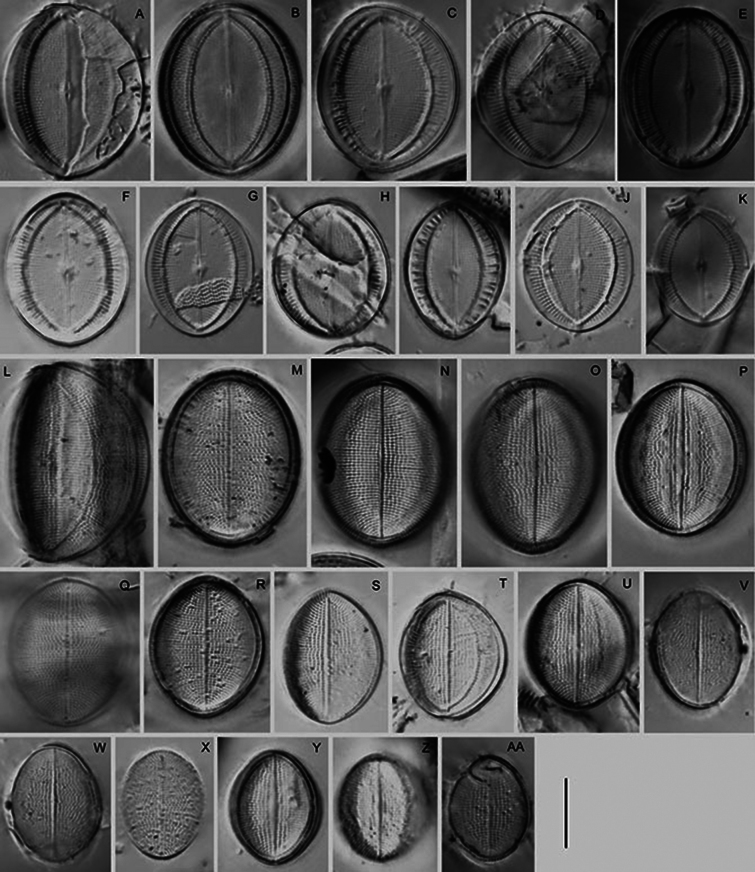
*Cocconeisbaicalensis*. LM. Lectotype. Slide No. 318a, Kotelnikovskiy Mayak, LE A0004242 **A–K** raphe valve **L–AA** sternum valve. Scale bar: 10 µm.

**Figure 4.**
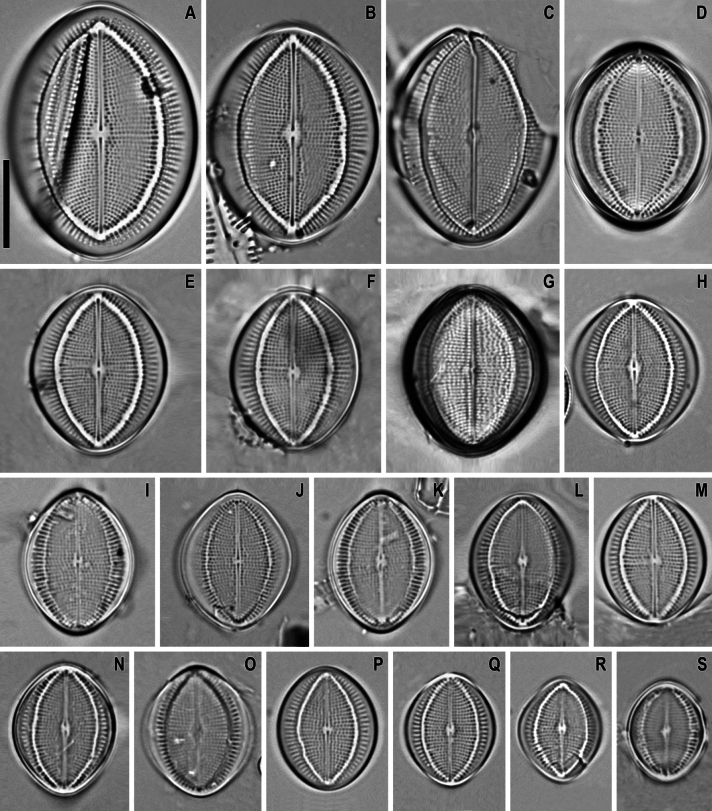
*Cocconeisbaicalensis*. Raphe valve. LM. Slides No. BL18566–BL18675, Island Bolshoy Ushkaniy. Scale bar: 10 µm.

**Figure 5.**
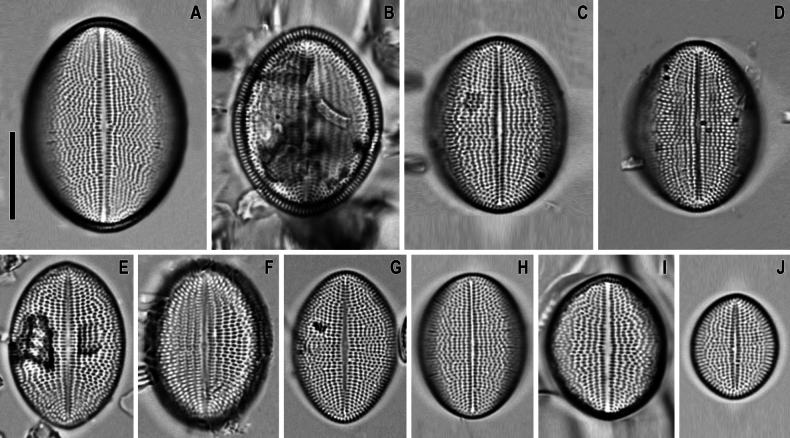
*Cocconeisbaicalensis*. Sternum valve. LM. Slides No. BL18566– BL18675, Island Bolshoy Ushkaniy. Scale bar: 10 µm.

**Figure 6.**
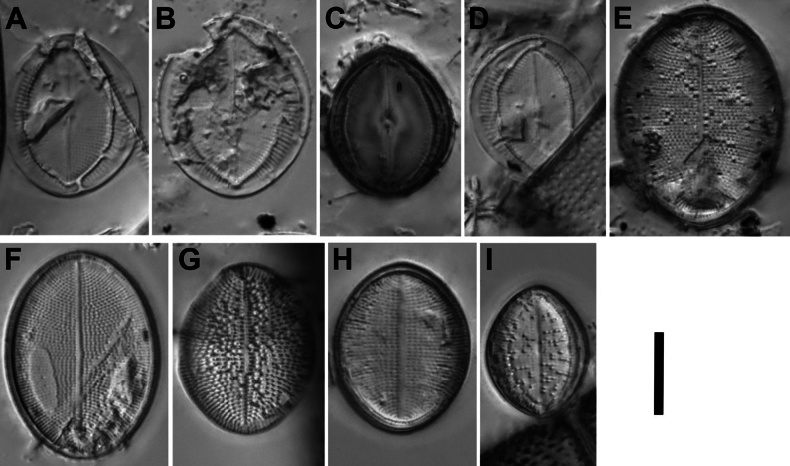
*Cocconeisbaicalensis*. LM. Slide No. 336a, Boguchanskaya Gulf. LE A0004244 **A–D** raphe valve **E–I** sternum valve. Scale bar: 10 µm.

**Figure 7.**
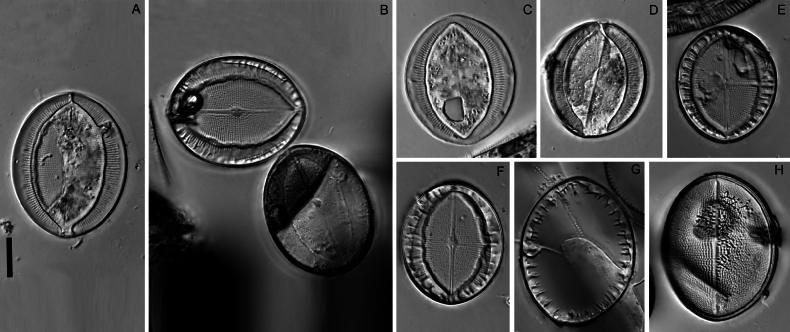
*Cocconeisbaicalensis*. LM. Slide No. 5, Marituy, LE A0002317 **A, D** sternum valve with valvocopula **B, C, E, F** raphe valve **G** valvocopula of raphe valve **H** sternum valve. Scale bar: 10 µm.

LM (n = 56) (Figs 3–7). Valves broadly elliptical, 11.2–29.2 μm in length (mean 19.4), 8.6–21.5 in breadth (mean 14.6). RV with straight raphe, SV with straight or slightly curved axial area. On RV 20–28 striae in 10 μm (mean 23.4) and 18.5–27.5 areolae in 10 μm of stria (mean 24.5), on SV 22–28 striae in 10 μm (mean 24.3) and 13.0–19.5 areolae in 10 μm of stria (mean 16.6). Length to breadth ratio 1.2–1.5:1 (mean 1.3:1).

SEM (n = 74). Valves from subcircular to broadly elliptical, with broadly rounded apices, 11.5–36.7 μm in length (mean 19.1–21.3), 8.7–27.1 in breadth (mean 15.2–17.2). Length to breadth ratio 1.0–1.4:1 (mean 1.2–1.3:1). RV and SV very different on structure.

RV (n = 28). Valves concave, with straight raphe and narrow-linear axial area. Striae uniseriate, 21–32 in 10 μm (mean 24.6), weakly radial in valve middle, more radial towards valve apices, interrupted by submarginal hyaline strips and not reached valve margin. Areolae 20–31 in 10 μm of stria (mean 24.8), absent at valve apices. Valve mantle reversed (contra mantle): valve face flat, mantle (or part of it) elevated and opposite directed to the adjacent SV, these two parts (flat and raised) separated from each other by narrow submarginal hyaline strips, 0.3–0.8 μm in breadth. Wider marginal ridge, 0.8–2.0 μm in breadth, at mantle or along valve edge.

**Figure 8.**
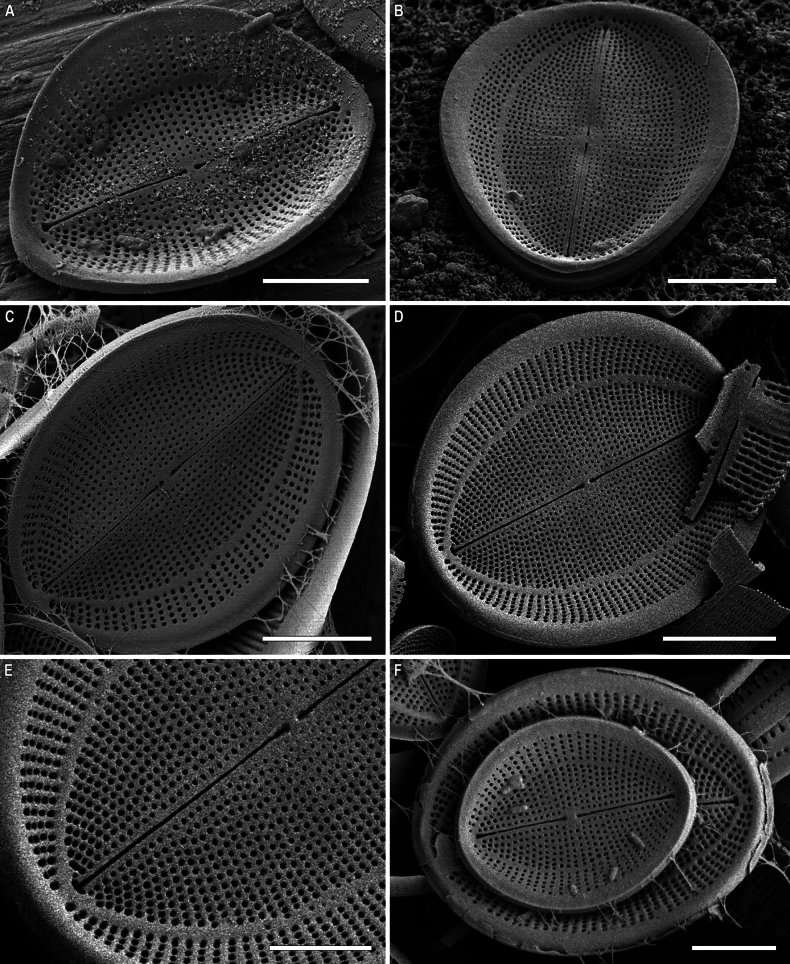
*Cocconeisbaicalensis*. Raphe valve, external view. SEM. Specimen No. BL15645, Island Bolshoy Ushkaniy. Scale bar: 5 µm.

RV, external view (Figs 8A–F, 11A–D, 13A, B, 15A, C, D). Central area small, round or rectangular. Raphe straight, proximal (central) endings drop-shaped, slightly extending into central area; distal endings more extending and frequently T-shaped. Areolae round or unevenly rounded, small along raphe, increasing towards valve edge.

**Figure 9.**
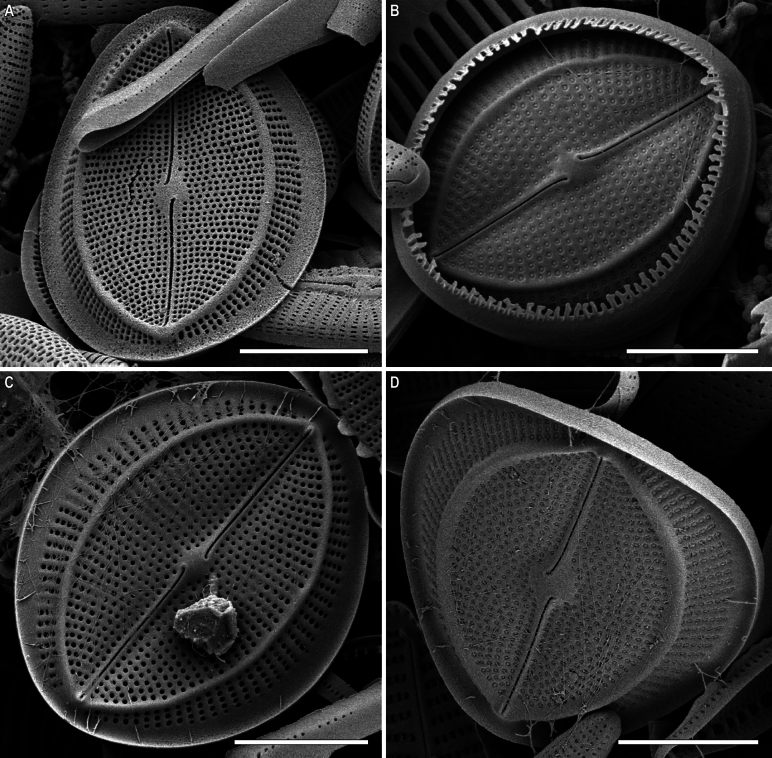
*Cocconeisbaicalensis***A–D** raphe valve, internal view **B** valve with valvocopula. SEM. Specimen No. BL15645, Island Bolshoy Ushkaniy. Scale bar: 5 µm.

RV, internal view (Figs 9A–D, 11E, F, 13C, 15B). Central area round, axial and central areas slightly convex. Proximal raphe endings directed opposite; distal ones straight or directed opposite from each other and from proximal endings, i.e. raphe branches S-shaped. Hyaline area (submarginal hyaline strips) in form of ridge. Areolae round, equal in size, and occluded (not always) by hymen.

RVVC (Fig. 13D). Valvocopula connected conically to RV, closed or opened, up to 3 μm in breadth, with uneven edge bears and elongated weakly curved fimbriae, 0.9–1.8 μm high, 13–18 in 10 μm, some fimbriae with dichotomously branched tips.

SV (n = 45). Valves convex, increasing of convexity closer to valve edge. Central area and hyaline rings absent. Striae uniseriate, 17–28 in 10 μm (mean 25). Areolae round to unevenly rounded, sometimes elongated, 11–23 in 10 μm of stria (mean 18.7).

SV, external view (Figs 12A–D, 14A, B, 15E). Axial area narrowly linear or absent. Middle part of valve (not mantle) often with small granules arranged in order or randomly.

**Figure 10.**
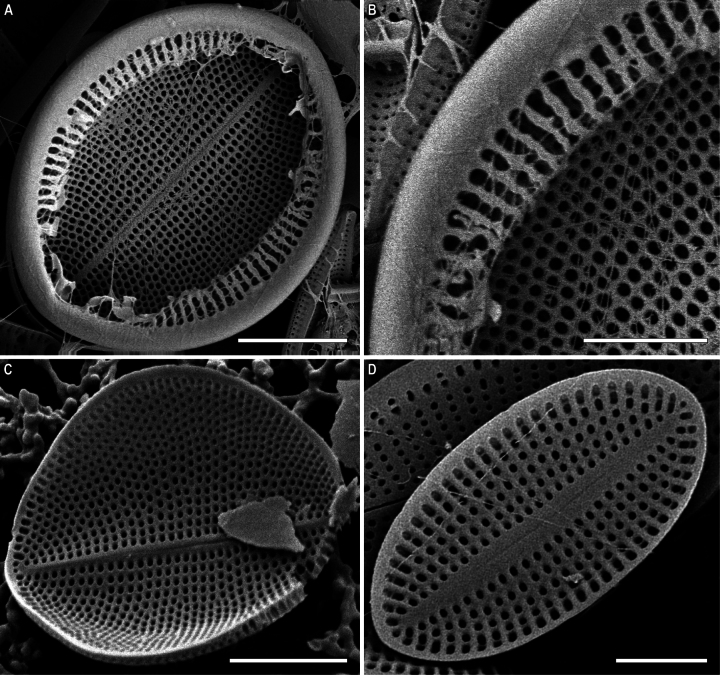
*Cocconeisbaicalensis***A–D** sternum valve, internal view **A, B** valve with valvocopula **D** lowest valve (arrowhead). SEM. Specimen No. BL15646, Island Bolshoy Ushkaniy. Scale bars: 5 µm.

SV, internal view (Figs 10A–D, 12E, F, 14C, D, 15F). Axial area narrowly linear to narrowly lanceolate, usually slightly raised as rib.

**Figure 11.**
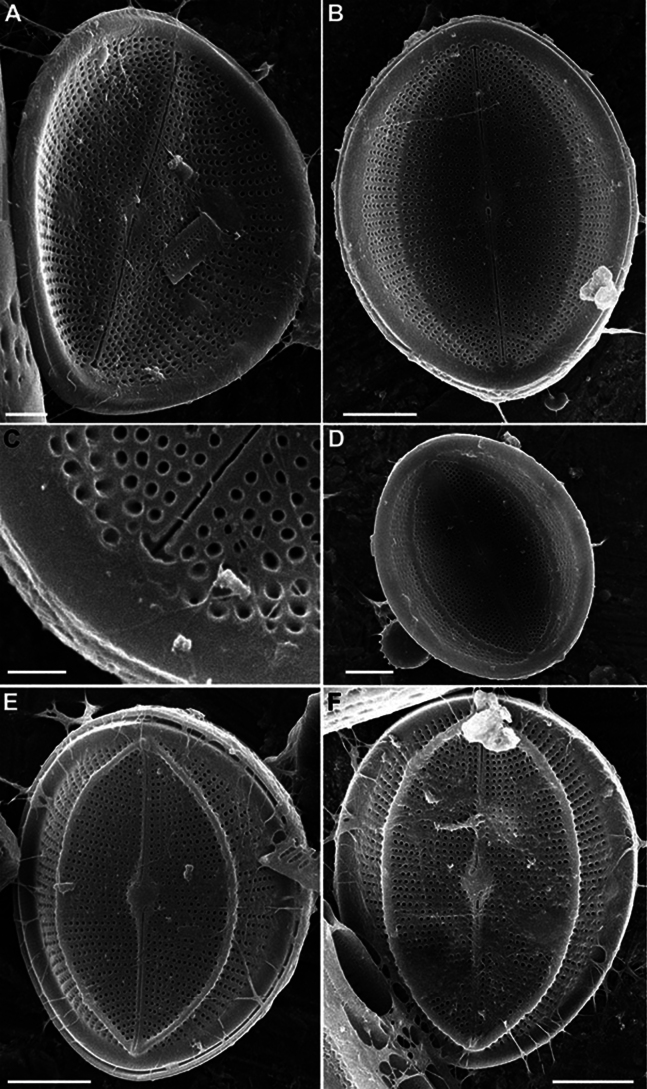
*Cocconeisbaicalensis*. Raphe valve (**A–D**) external view (**E, F**) internal view. SEM. Specimen No. 318, Kotelnikovskiy Mayak. Scale bars: 2 µm (**A**); 5 µm (**B, D–F**); 1 µm (**C**).

**Figure 12.**
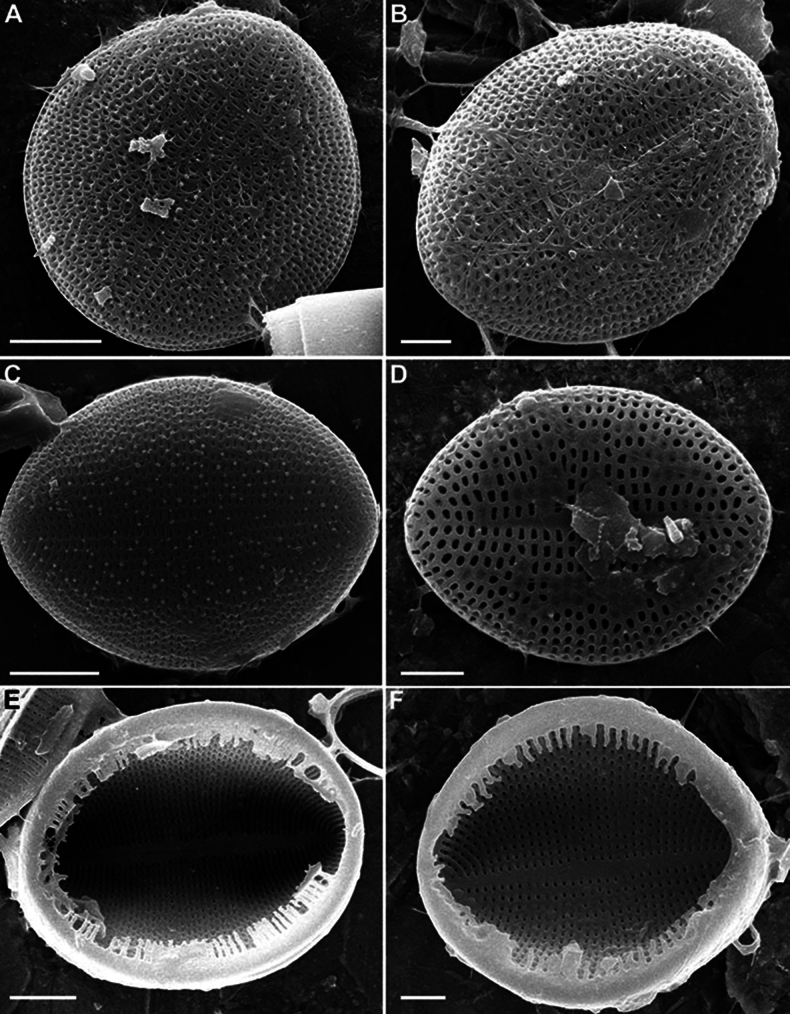
*Cocconeisbaicalensis*. Sternum valve (**A–D**) external view (**E, F**) internal view. SEM. Specimen No. 318, Kotelnikovskiy Mayak. Scale bars: 5 µm (**A, C, E**); 2 µm (**B, D, F**).

**Figure 13.**
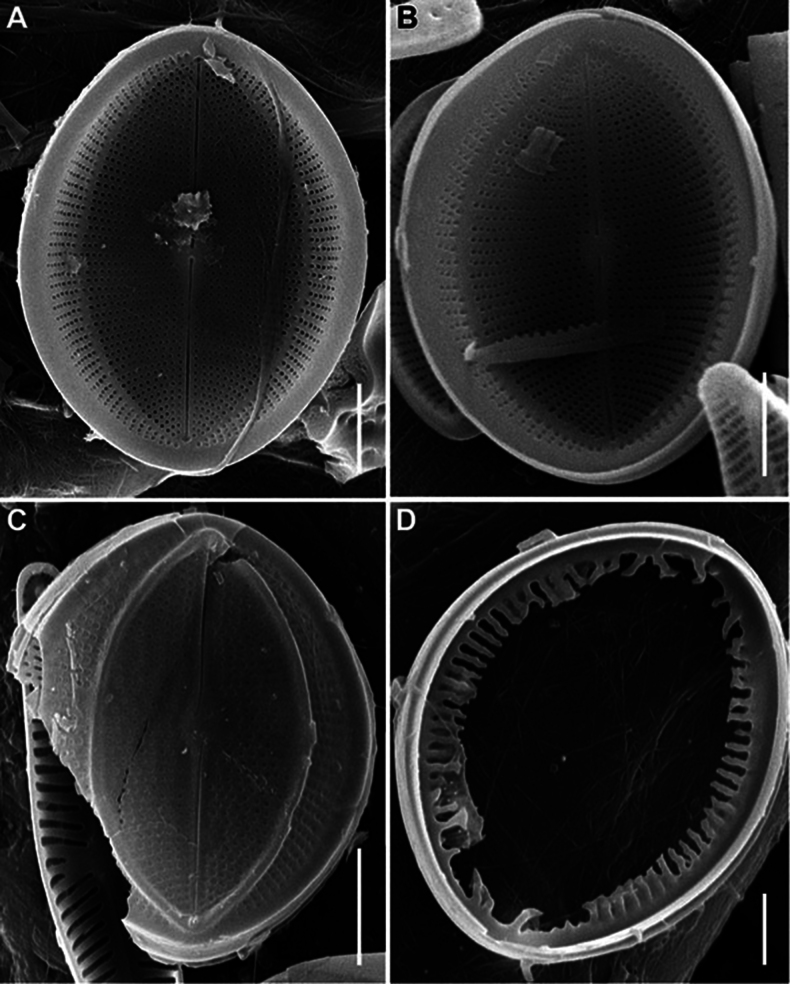
*Cocconeisbaicalensis***A–C** raphe valve **A, B** external view **C** internal view **D** valvocopula (VCRV). SEM. Specimen No. 5, Marituy. Scale bars: 5 µm (**A–C**); 2 µm (**D**).

**Figure 14.**
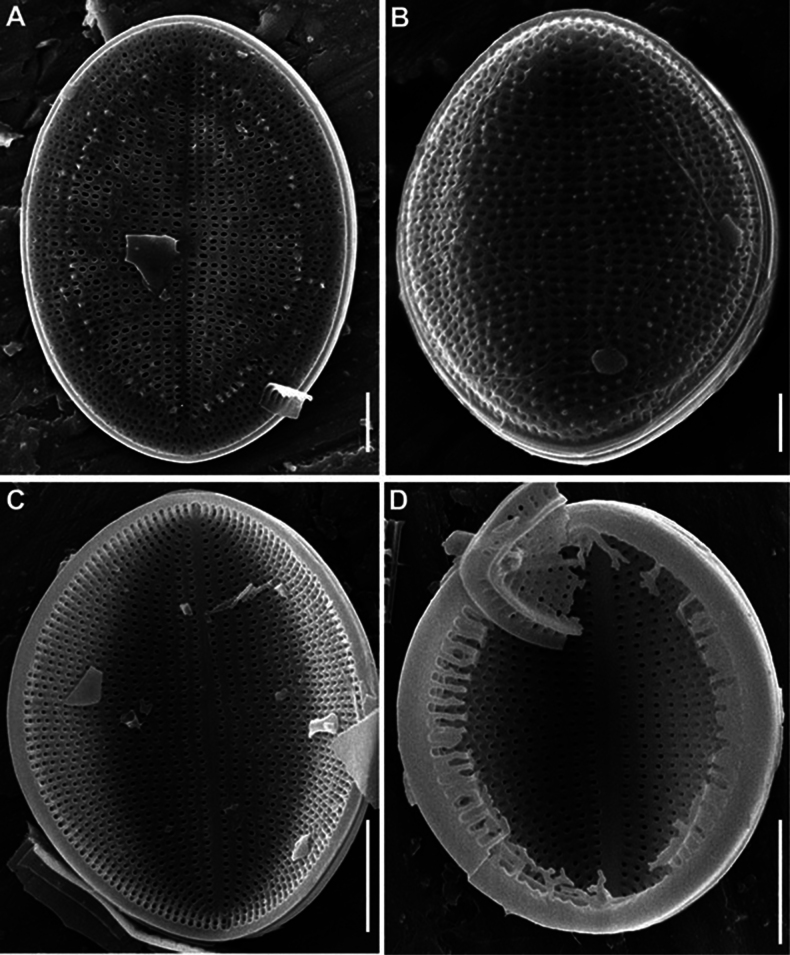
*Cocconeisbaicalensis***A–D** sternum valve **A, B** external view **C** internal view **D** valve with valvocopula. SEM. Specimen No. 5, Marituy. Scale bars: 5 µm (**A, C, D**); 2 µm (**C**).

**Figure 15.**
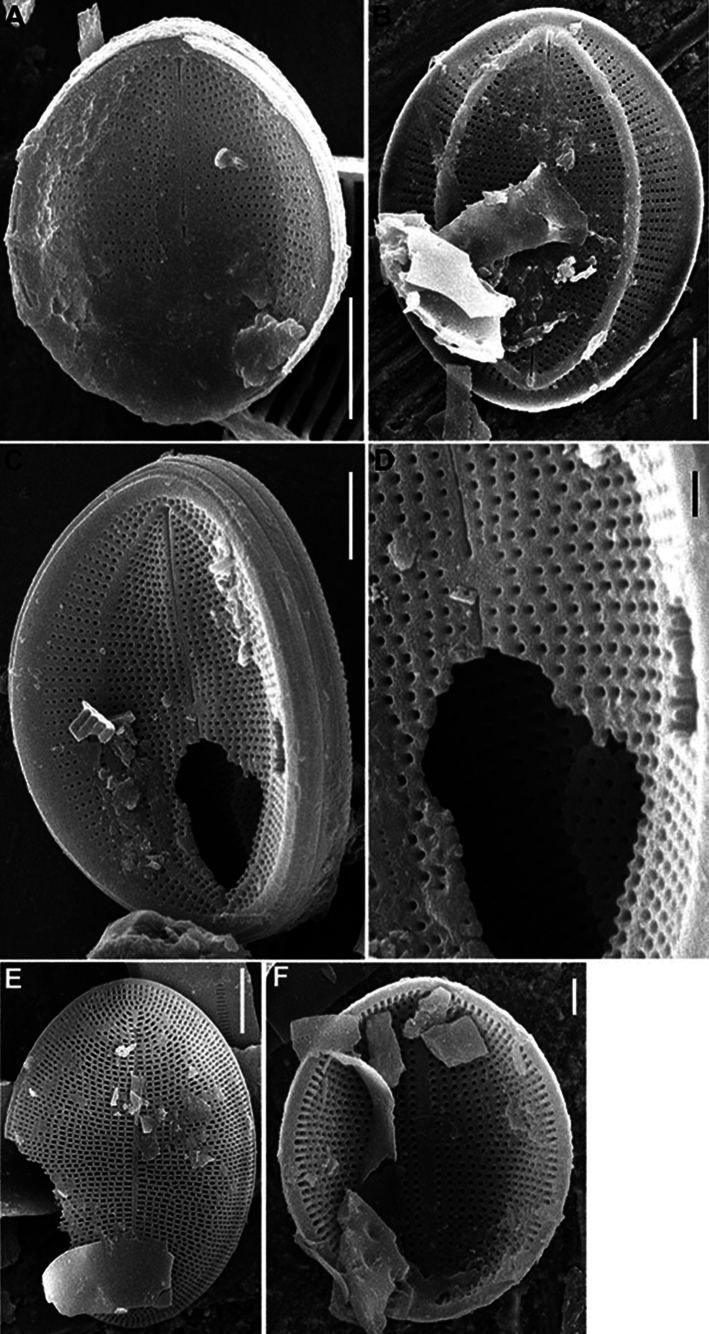
*Cocconeisbaicalensis***A, B** raphe valve **A** external view **B** internal view **C** frustule **D** part of frustule, external RV and internal SV view **E, F** sternum valve **E** external view **F** internal view. SEM. Specimen No. 336, Boguchanskaya Gulf. Scale bars: 5 µm (**A–C, E**); 1 µm (**D**); 2 µm (**F**).

SVVC. Valvocopula closed, 1.3–1.7 μm in breadth.

There are no data on copulae, except valvocopulae.

#### Comments.

Our data differ from the data of Skvortzov and Skabichevsky in the following (Table 4): the valve length corresponds to Skvortzov’s description (Skvortzov 1946). The upper range of valve width and the stria density on both valves are increased. The protologue data of 14 striae in 10 μm were not observed and are likely erroneous. Literature data on the areola density in striae most likely refer to a SV, according to our data it is somewhat higher than it is in the literature; while the areola density on the RV is significantly higher than on the SV.

**Table 4.** Comparison of morphological features of *Cocconeisskvortzowii* and related species.

Species	Valve length (μm)	Valve breadth (μm)	Ratio	Striae number in 10 μm		Areolae number in 10 μm	Distribution	References
				RV	SV			
*Cocconeisskvortzowii*	8.9–27.0	5.6–17.7	1.3–2.0:1	20–24	13–23	22–33 (RV)	Lake Baikal	Our data
C.placentulavar.skvortzowii	11–36	6.8–22.0	1.6–1.8(2.1):1*	20–30	13–20	11–13 (SV)	Lake Baikal	Skvortzow 1937; Skabichevsky 1977
C.placentulavar.skvortzowii	12–24	7–14	1.7:1*	~30	18	n.d.	Lake Baikal	Sheshukova 1950
*C.skvortzowii*	14–36	8–22	1.6–1.8:1*	15–20	15–20	11–13	Lake Baikal	Sheshukova 1951
*C.disculus* (Schumann) Cleve	20–25	11–16	1.6–1.8:1*	22	7–9	n.d.	Europe	Sheshukova 1950, 1951
	10–25	7–16	1.4–1.6:1*	20–22	6–10	18–22 (RV)	Cosmopolitan, Germany	Krammer and Lange-Bertalot 1991; Hofmann et al. 2011; Kulikovskiy et al. 2016b; Lange-Bertalot et al. 2017
*C.diminuta* Pant.	8.5	6	1.4:1*	35	24	n.d.	Lake Baikal	Skvortzow 1937
C.disculusvar.diminuta (Pant.) Scheshukova	7–15	5–9	1.4–1.7:1*	~32	13	n.d.	European Russia, Mongolia	Sheshukova 1950, 1951
*C.neodiminuta* Krammer	7–18	5–9	1.4–2:1*	24–32	11–14 (2–4 areolae per stria)	25–32 (RV)	Germany, Hungary, Switzerland, Ireland	Krammer and Lange-Bertalot 1991; Kulikovskiy et al. 2016b
	9.6–19.4	6.5–8.0	1.5–2.4:1*	ca. 25	n.d.	n.d.	Poland	Wojtal 2009
	8–18	6–9	1.3–2:1*	24–32	11–14	25–32 (RV)	Germany, Hungary, Ireland	Romero and Van de Vijver 2011
	13–17	7–10	1.7–2:1*	30	13–15	n.d.	Brazil	Costa et al. 2020
C.placentulavar.euglypta (Ehrenb.) Grunow	10–46	n.d.	n.d.	n.d.	19–22	n.d.	Cosmopolitan	Krammer and Lange-Bertalot 1991
	11.0–41.2	7.4–26.6	1.5:1*	ca. 20	20–22	n.d.	Poland	Wojtal 2009
*C.euglypta* Ehrenb. (epitype)	15.9–29.5	9.8–17.7	1.5–1.8:1	17–22	18.5–24 / 20–24	n.d.	Baltic Sea	Romero and Jahn 2013
	15–30	9–18	1.7:1*	17–22	18–24	n.d.	Cosmopolitan	Kulikovskiy et al. 2016b
	15–45	9–28	1.5–1.8:1	17–22	18–24	n.d.	n.d	Lange-Bertalot et al. 2017
	13.4–29.5	8.3–16.6	1.6–1.8:1*	19–24	22–24	n.d.	Brazil	Costa et al. 2020
C.placentulavar.lineata (Ehrenb.) Van Heurck	30	18	1.7:1*	n.d.	24	n.d.	Lake Baikal	Skvortzow 1937
	40–70	30–40	1.3–1.8:1*	n.d.	n.d.	n.d.	Russia, Europe	Sheshukova 1951
	10–80	n.d.	n.d.	n.d.	16–23	n.d.	Cosmopolitan	Krammer and Lange-Bertalot 1991
	14.0–25.2	10.5–14.0	1.3–1.8:1*	22.0–23.5	20–22	n.d.	Poland	Wojtal 2009
	11–42	7–28	1.5–1.6:1*	15–24(30)	n.d.	n.d.	North Russia	Genkal and Vekhov 2007
*C.lineata* Ehrenb. (epitype)	18.6–22.7	6.4–13.1	1.6–2.4:1	20–28	(7)10–15 / 12–20	16–24(26) (SV)	Faroer Islands, Denmark	Romero and Jahn 2013
*C.lineata*	18–23	6–14	1.6–3:1*	20–28	10–15	16–24 (SV)	Cosmopolitan	Kulikovskiy et al. 2016b
	16–80	6–35	1.6–2.4:1	20–28	10–15	16–24 (SV)	Europe	Lange-Bertalot et al. 2017
	12.7–19.6	7.9–10.9	1.6–1.8:1	21–22	25–32	n.d.	Brazil	Costa et al. 2020
	19.1–22.8	9.4–12.6	1.6–2.2	18–24	22–28	25–28 (RV), 12–18 (SV)	Korea	Jahn et al. 2017
C.placentulavar.pseudolineata Geitler	7.5–38.0	n.d.	n.d.	n.d.	13–20(22)	n.d.	n.d.	Krammer and Lange-Bertalot 1991
	7.5–38.0	n.d.	n.d.	16–20(22)	20–23	n.d.	n.d.	Romero and Van de Vijver 2011
*C.pseudolineata* (Geitler) Lange-Bert.	7.5–38.0	6–18	1.3–2.1:1*	20–23	13–18(20)	16–20(22) (SV)	Europe	Hofmann et al. 2011; Lange-Bertalot et al. 2017
	12.5–23.0	8–18	1.3–1.6:1*	23–24	13–15	n.d.	Poland	Wojtal 2009
	16–32	8–14	2–2.3:1*	23–26	12–22	23–28 (RV)	n.d.	Werum and Lange-Bertalot 2004
*C.pseudothumensis* Reichardt	11.5–13.5	8.2–9.5	1.4:1*	35–40	10–20	10–20 (RV)	Europe, France, Germany, Switzerland	Reichardt 1982; Romero and Van de Vijver 2011
	11.5–13.5	8.2–9.5	1.4:1*	35–40	10–12	n.d.	France, Europe	Krammer and Lange-Bertalot 1991; Hofmann et al. 2011; Lange-Bertalot et al. 2017
	9–15	6.5–11.0	1.4:1*	35–40	10–14	n.d.	Holarctic	Kulikovskiy et al. 2016b
*C.thumensis* Ant. Mayer	Up to 10	5–6	n.d.	15–16	15–16	n.d.	North European Russia, Europe	Sheshukova 1950, 1951
*C.neothumensis* Krammer	6.5–13.0	4.0–8.3	1.6:1*	28–36	16–25	34–37 (RV)	Cosmopolitan, Germany	Krammer and Lange-Bertalot 1991; Hofmann et al. 2011; Kulikovskiy et al. 2016b; Lange-Bertalot et al. 2017
	11.2–12.2	7.0–7.4	1.6:1*	23	26	n.d.	Brazil	Costa et al. 2020
	8.0–10.6	4.4–8.0	1.3–1.8:1*	25–30	28	n.d.	North Russia	Genkal and Vekhov 2007

* – our calculations on illustrations; n.d. – no data.

*Cocconeisbaicalensis*, in contrast to *C.placentula* Ehrenberg, has (1) broadly elliptical valves, (2) the lanceolate submarginal hyaline strip on the RV, (3) the stria density is higher on the RV and lower on the SV, (4) striae located along the valve edge are interrupted on/near the apices by the submarginal hyaline strips, (5) rounded areolae on the SV, (6) the sternum on the SV is clear on the internal surface.

We choose SEM photograph Fig. 9C, as the epitype of *C.baicalensis*, to show the morphological features of the RV in the internal view, which are poorly represented in the type material, namely its 3D-shape, density and arrangement of striae, shape and size of hyaline areas. Additionally, figures 8A, 10A and 12C show the same traits of the RV in the external view and SV in the external and internal view.

The validating description of Cocconeisplacentulavar.baikalensis (Williams and Reid 2001, 310) (≡*Cocconeisskvortzowii*) is “based on a little bottom sample collected by Prof. K.I. Meyer at the depth of 33 meters near the Olhon Gate of Baikal Lake July 29, 1916” (Williams and Reid 2001, 297). No specimen of this gathering has been found in LE. In its absence, the only element of the original material are the illustrations in the protologue (Williams and Reid 2001, pl. 5, figs 5, 7, 8), two of which we are designating as the lectotype.

Since the drawings (Williams and Reid 2001, pl. 5, figs 7, 8) do not demonstrate the morphological details, but only the valve outlines and a wide axial area on both valves, we are designating the epitype, namely SEM photograph Fig. 18C of Skabichevsky’s material from the Island Bolchoy Ushkaniy. The epitype illustration clearly shows the morphological features of the RV and SV in the external and internal view, namely shape, density and arrangement of striae, shape and size of areolae.

### ﻿ Cocconeis skvortzowii

Taxon classificationPlantaeCocconeidalesCocconeidaceae

(Sheshukova) Sheshukova 1951 in Proshkina-Lavrenko (ed.) Opredelitel presnovodnykh vodoroslei SSSR 4: 193, figs 104 a–c (with indirect reference; with authorship of basionym “Skv.”) emend. Gogorev & Yurchak

69A3A1EC-76E0-563F-A880-815F214FB309

Cocconeis placentula Ehrenberg var. skvortzowii Skabichevskij ex Sheshukova 1950 in Proshkina-Lavrenko (ed.) *Diatomovyi analiz* 3: 86, pl. 30, figs 10 a, b (with indirect reference; as combination with authorship “(Skv.) Skabitsch.”). Basionym.
Cocconeis placentula Ehrenberg var. baikalensis Skvortzov 1937 in *Philippine Journal of Science*, *C* 62(3): 310, pl. 5, figs 5, 7, 8, illegitimate name, later homonym of C.placentulavar.baicalensis Skvortzov & K.I. Meyer 1928. Replaced synonym.
≡ CocconeisplacentulaEhrenbergvar.sibirica Skabichevskij 1952 in *Botanicheskie materialy otdela sporovykh rasteniy Botanicheskogo instituta imeni V.L. Komarova* 8: 36 (as combination with authorship of basionym “Skv.”), illegitimate superfluous name. ≡ CocconeisplacentulaEhrenbergsubsp.sibirica Skabichevskij 1977 in *Prirodnye kompleksy nizshikh rastenii Zapadnoi Sibiri*: 127, fig. 2, 9–11 (as combination with authorship of basionym “Skv.”). Synonyms.

#### Type materials.

***Lectotype*** • (designated here): Skvortzov, 1937, Philippine Journal of Science, Section C 62(3): 310, Pl. 5, figs 7, 8.

***Epitype*** • (designated here): figures here represented by Fig. 18C (Baikal, specimen BL15645, leg. A.P. Skabichevsky, 20 July 1965).

#### Type locality.

Russia, Siberia, Lake Baikal, Olhon Gate, Boguchanskaya Gulf, Cape Elokhin, Cape Kotelnikovskiy.

#### Description.

**Figure 16.**
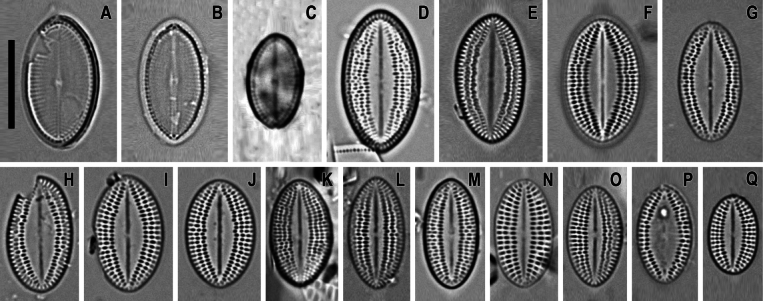
*Cocconeisskvortzowii***A–C** raphe valve **D–Q** sternum valve **D–O, Q** valve with ghost raphe. LM. Slides No. BL18566– BL18675, Island Bolshoy Ushkaniy. Scale bar: 10 μm.

LM (n = 17) (Fig. 16A–Q). Valves lanceolate-elliptical, 8.9–16.1 μm in length (mean 12.7), 5.6–9.0 μm in breadth (mean 7.1). Length to breadth ratio 1.5–2.0:1 (mean 1.8:1). RV with straight raphe, SV with broad lanceolate axial area. On RV 20–24 striae in 10 μm (mean 22), on SV – 18–23 in 10 μm (mean 21.4). Often SV with “ghost raphe”, so such valves similar to RV in view. Valvocopula closed or open, with sparsely spaced fimbriae.

SEM (n = 17). Valves from elliptical to linear-elliptical, 11.6–27.0 μm in length (mean 14.7–16.7), 6.3–17.7 in breadth (mean 9.1–10.5). Length to breadth ratio 1.3–1.9:1 (mean 1.6:1).

RV (n = 9). Valves concave, with slightly convex middle part. Axial area narrowly linear or indistinguishable. Striae uniseriate, 21–24 in 10 μm (mean 22.7). Areolae rounded or unevenly rounded, 22–33 in 10 μm of stria (mean 29.1), absent at valve apices. Valve mantle (contra mantle) slightly reversed.

**Figure 17.**
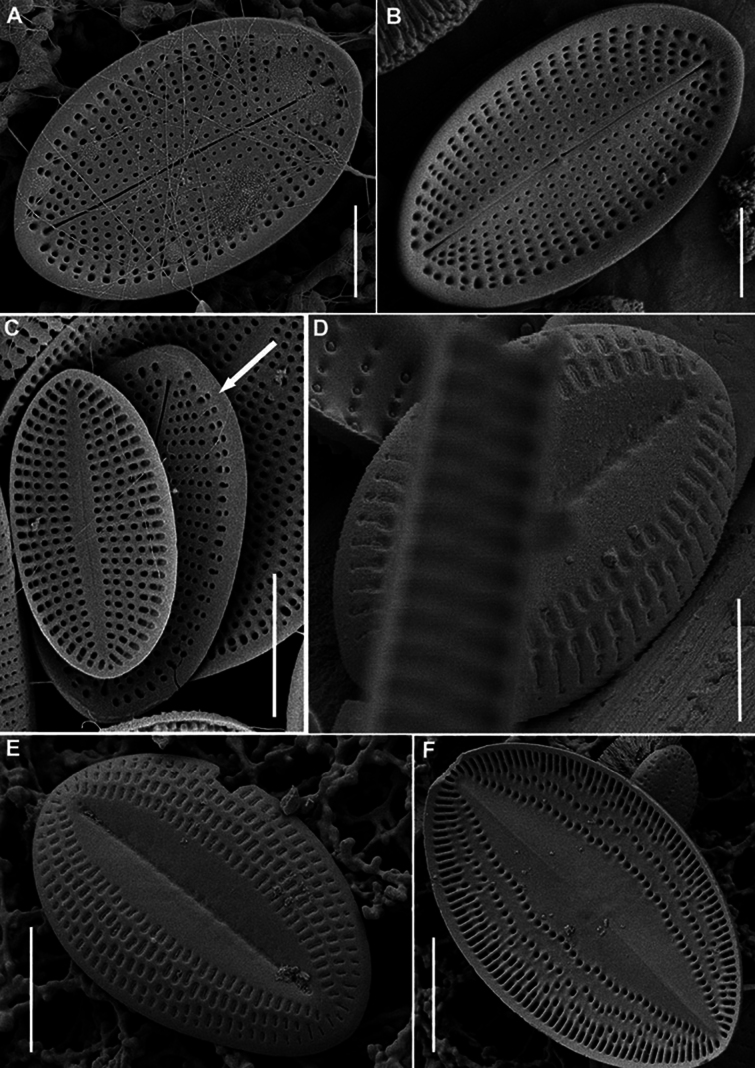
*Cocconeisskvortzowii***A–C** raphe valve, external view **C** Lower valve (arrowhead) **D–F** sternum valve **D, E** external view **F** internal view. SEM**A, C** specimen No. BL15646, Island Bolshoy Ushkaniy **B, D–F** specimen No. BL15645, Island Bolshoy Ushkaniy. Scale bars: 3 µm (**A, B, D**); 5 µm (**C**); 6 µm (**E**); 8 µm (**F**).

RV, external view (Figs 17A–C, 19A–C). Central area absent. Raphe straight, proximal endings slightly widened, distal endings more widened. Striae weakly radial, sometimes interrupted by clear or weakly defined narrow submarginal hyaline strips. Areolae increased in size and more densely located toward valve edge. Marginal ridge wider, 0.4–1.0 μm in breadth, and more distinct than submarginal hyaline strips.

RV, internal view (Fig. 19D). Central area small, round. Proximal raphe endings directed opposite, distal endings form small helictoglossae. Areolae equal in size.

RVVC. Valvocopula closed, up to 1.3 μm in breadth, with straight or weakly curved fimbriae, up to 1.3–1.6 μm high, 7–8 in 10 μm.

SV (n = 8). Valves convex, with concave middle part. Axial area usually wide, lanceolate, rarely narrowly or broadly lanceolate, with often ghost raphe. Central area absent. Striae uniseriate, 13–20 in 10 μm (mean 17.6), consisted of 2–4 elongated areolae.

**Figure 18.**
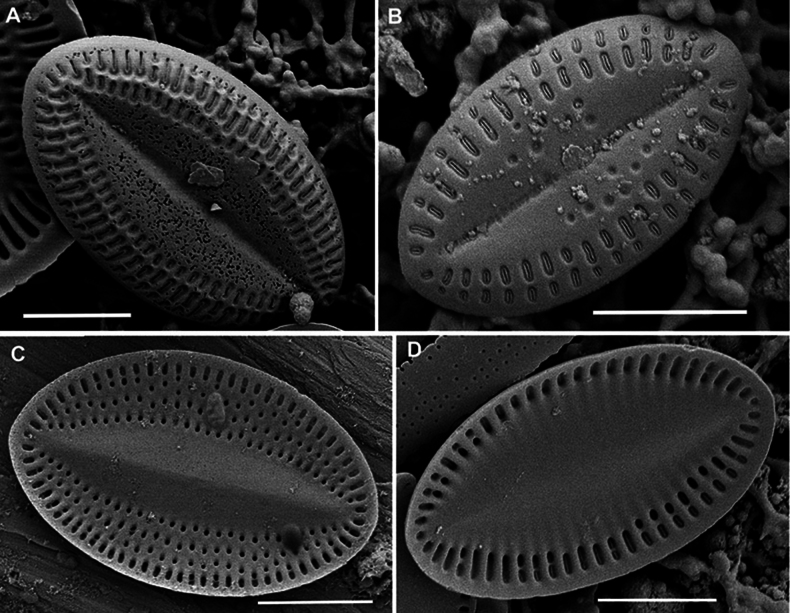
*Cocconeisskvortzowii***A–F** sternum valve **A, B** external view **B** sternum with ghost raphe and ghost areolae **C, D** internal view. SEM. Specimen No. BL15645, Island Bolshoy Ushkaniy. Scale bars: 4 µm (**A, B, D**); 5 µm (**C**).

SV, external view (Figs 17D, E, 18A, B). Openings of areolae slit-like, usually located in depression. Axial area often with ghost areolae (small depressions) located in “striae” or randomly.

**Figure 19.**
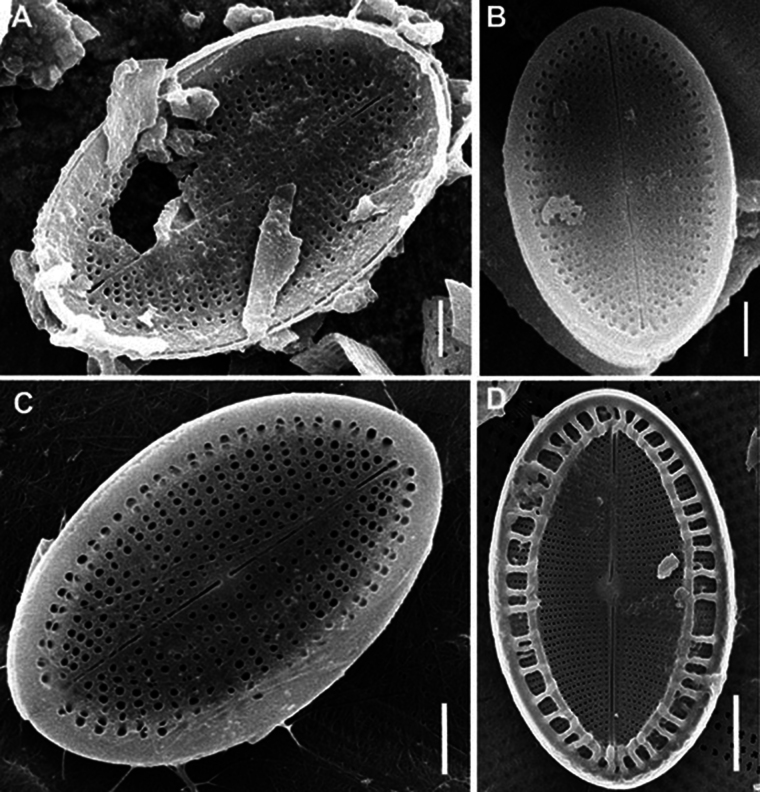
*Cocconeisskvortzowii***A–D** raphe valve **A–C** external view **D** valve with valvocopula, internal view. SEM**A, B** specimen No. 336, Boguchanskaya Gulf **C** specimen No. 318, Kotelnikovskiy Mayak **D** Cape Elokhin. Scale bars: 2 µm (**A–C**); 5 µm (**D**).

SV, internal view (Figs 17F, 18C, D). At edge, striae consisted of 1–2 rounded or elongated areolae, divided into 1–2 parts by thin or thickened baffle. Areolae rounded or irregularly rounded near axial area, in 2–3 (up to 5) curving apical rows (distance between areolae in stria different). Adjacent “postmacroareolae” delimited by small ribs and sometimes extended up to axial area.

There are no data on copulae, except valvocopulae.

#### Comments.

Our data differ from the data of Skvortsov and Skabichevsky (Table 4) in the following: the range of valve length and width has been increased, namely, the minimal size of the studied valves is less than those noted in the literature; the stria density on the RV corresponds to the previous data, but density on the SV has been increased. Data in the literature available on areola density in stria most likely refer to a SV; our data obtained are significantly higher and refer to a RV.

Valves shown in Figs 17D, E, 18A, B are somewhat similar to *Cocconeismargaritata* Riaux-Gobin & Al-Handal and *Cocconeis* spp. (Riaux-Gobin et al. 2010) according to the areolae pattern on external surface of the SV, namely, the openings of areolae are slit-like and located in a depression.

A comparison of *Cocconeisskvortzowii* with related species (Table 4) showed that the species is externally similar to small valves of *C.pseudothumensis* (including the ratio of valve length to breadth), but differs greatly in the stria density on the RV. The same results can be seen for *C.neothumensis* as well as the *C.diminuta* / *C.neodiminuta* complex.

The comparative analysis shows similarity/overlapping in stria density of *Cocconeisskvortzowii* with *C.lineata* and *C.pseudolineata*. But two last species have the larger size of valve compared to *C.skvortzowii*, and, accordingly, a larger length-to-breadth ratio. Data on the stria density on the SV in *C.lineata* are quite contradictory from different references, which most likely indicates a complex of several species. If we take into account only data on the epitype of *C.lineata*, then their stria density is lower than that of *C.skvortzowii*.

Two species, *Cocconeisdisculus* and *C.euglypta*, differ from *C.skvortzowii* in both valve shape and SV structure, namely, in the number of apical rows of areolae.

A direct reference to the location of CocconeisplacentulaEhrenb.var.baicalensis is absent in Skvortzow and Meyer (1928): “The list is published without reference to the place where a certain species had been found”. However, the locations of 36 samples are given in their publication, of which later Meyer (1930, 341) indicated 8 typical locations for this variety (Table 1).

The three permanent slides with the date given in the protologue, and one more with a later date are deposited in the LE diatom collection. Slides No. 15 and 16 (according to the numbering of Skvortzow and Meyer 1928, 2) are labelled as the village of Merkusheva (“village Merkutov”), on the label of third one it is given “d. Merkushevo [Merkusheva].” The exact location (with coordinates) was established using a 1912 map (http://www.etomesto.ru/map-chita_zabaykalskaya-oblast-1912). Reliable and abundant findings of *Cocconeisbaicalensis* were not identified when studying these slides in LM.

Also, in the Diatom collection LE there is authentic material collected by K.I. Meyer in 1921–1928 (35 samples, Table 1), including 9 samples (data June 30 1926, or no data), the locations of which are indicated in Skvortzow and Meyer (1928) and Meyer (1930). The last ones are used to make permanent slides stored in LE, in two of them (LE A0004242–LE A0004245) clear and numerous valves of *Cocconeisbaicalensis* were found.

Skabichevsky’s material (Table 1) was studied in LM and SEM to clarify the morphology and taxonomy of *Cocconeisbaicalensis*, since many morphological characters and elements of the valve are not satisfactory when studied in LM. We choose SEM photograph Fig. 9C, as the epitype of *C.baicalensis*, which clearly show the morphological features of the RV in the internal view, namely its 3D-shape, density and arrangement of striae, shape and size of hyaline areas, poorly represented in the type material. Additionally, figs 8A, 10A, 12C show the same of the RV in the external view and SV in the external and internal view.

## Supplementary Material

XML Treatment for Cocconeis baicalensis

XML Treatment for Cocconeis skvortzowii

## ﻿Additional information

### Author ORCIDs

Rinat Gogorev https://orcid.org/0000-0001-8668-7912

Maria Yurchak https://orcid.org/0000-0002-6079-539X

Irina Sokolova https://orcid.org/0000-0001-5277-6669

Anton Glushchenko https://orcid.org/0000-0002-2832-1872

Maxim Kulikovskiy https://orcid.org/0000-0003-0999-9669

### Data availability

All of the data that support the findings of this study are available in the main text.

## References

[B1] Costa LF Wetzel CE Ector L Bicudo DC (2020) Freshwater *Cocconeis* species (Bacillariophyceae) from Southeastern Brazil, and description of *C.amerieuglypta* sp. nov. Botany Letters 167(1): 15–31. 10.1080/23818107.2019.1672103

[B2] Genkal SI Vekhov NV (2007) Diatoms of waterbodies in the Russian Arctic: Novaya Zemlya archipelago and Vaigach Island; Moscow, Russia, 64 pp. [In Russian]

[B3] Gogorev RM Chudaev DA Stepanova VA Kulikovskiy MS (2018) Russian and English terminological glossary on morphology of diatoms. Novosti Sistematiki Nizsih Rastenij 52(2): 265–309. 10.31111/nsnr/2018.52.2.265

[B4] Gogorev RM Chudaev DA Gololobova MA (2024) Addition to the glossary of diatom morphology. Novosti Sistematiki Nizshikh Rastenii 58(1): A35–A58. 10.31111/nsnr/2024.58.1.A35

[B5] Gololobova MA (2012) Checklist of Boris V. Skvortzow’s diatom taxa. Iconographia Diatomologica 23: 611–742.

[B6] Guiry MD Guiry GM (2024) AlgaeBase. World-wide electronic publication, National University of Ireland: Galway. https://www.algaebase.org [accessed on 18 Aug 2024]

[B7] Hofmann G Werum M Lange-Bertalot H (2011) Diatomeen im Süßwasser — Benthos von Mitteleuropa. Bestimmungsflora Kieselalgen für die ökologische Praxis. Über 700 der häufigsten Arten und ihre Ökologie; Königstein, Germany, 908 pp.

[B8] Kociolek JP Stoermer EF (1988) Taxonomy and systematic position of the *Gomphoneisquadripunctata* species complex. Diatom Research 3(1): 95–108. 10.1080/0269249X.1988.9705019

[B9] Krammer K Lange-Bertalot H (1991) Bacillariophyceae. Teil 4. Achnanthaceae & Kritische Ergaenzungen zu *Navicula* (Lineolatae) und *Gomphonema*. Süsswasserflora von Mitteleuropa. Bd. 2/4. Stuttgart, Germany, 437 pp.

[B10] Kulikovskiy MS Lange-Bertalot H Metzeltin D Witkowski A (2012) Lake Baikal: Hotspot of endemic diatoms. I. Iconographia Diatomologica 23: 7–608.

[B11] Kulikovskiy MS Lange-Bertalot H Kuznetsova IV (2016a) *Cocconeisnanoburyatica* sp. nov. – a new monoraphid diatom species from Lake Baikal. Inland Water Biology 9(2): 112–115. 10.1134/S1995082916020103

[B12] Kulikovskiy MS Glushchenko AM Genkal SI Kuznetsova IV (2016b) Identification Book of Diatoms from Russia. Filigran, Yaroslavl, Russia, 804 pp. [In Russian]

[B13] Lange-Bertalot H Hofmann G Werum M Cantonati M (2017) Freshwater Benthic Diatoms of Central Europe: Over 800 Common Species Used in Ecological Assessment. Koeltz Botanical Books, Oberreifenberg, Germany, 942 pp.

[B14] Meyer K (1930) Einfuhrung in die Algenflora des Baicalsees. Bulletin of the Moscow Society of Naturalists, Biological section [Bulletin de la Société des Naturalistes de Moscou, section Biologique] 39: 179–396. [In Russian]

[B15] Reichardt E (1982) Diatomeen aus dem Lough Neagh. Nova Hedwigia 38: 401–420.

[B16] Riaux-Gobin C Romero OE Al-Handal AY Compère P (2010) Two new *Cocconeis* taxa (Bacillariophyceae) from coral sands off the Mascarenes (Western Indian Ocean) and some related unidentified taxa. European Journal of Phycology 45(3): 278–292. 10.1080/09670260903560076

[B17] Romero O Jahn R (2013) Typification of *Cocconeislineata* and *Cocconeiseuglypta* (Bacillariophyta). Diatom Research 28(2): 175–184. 10.1080/0269249X.2013.770801

[B18] Romero OE Rivera P (1996) Morphology and taxonomy of three varieties of *Cocconeiscostata* and *C.pinnata* (Bacillariophyceae) with considerations of *Pleuroneis*. Diatom Research 11(2): 317–343. 10.1080/0269249X.1996.9705388

[B19] Romero O Van de Vijver B (2011) *Cocconeiscrozetensis*, a new monoraphid diatom from Subantarctic freshwater and moss habitats. Diatom Research 26(1): 89–98. 10.1080/0269249X.2011.575118

[B20] Sheshukova VS (1950) Subordo Monoraphineae ... Diatomovyi analiz. Opredelitel iskopaemykh i sovremennykh diatomovykh vodoroslei. Kniga 3. Poryadok Pennales State Publishing House of Geological Literature, Leningrad, Russia, 78–116. [In Russian]

[B21] Sheshukova VS (1951) Podporyadok Monoraphineae – Odnoshovnye [Subordo Monoraphineae]. Opredelitel presnovodnykh vodoroslei SSSR. Vypusk 4. Diatomovye vodorosli. Sovetskaya Nauka, Moscow, 188–231. [In Russian]

[B22] Skabichevsky AP (1974) On the true authorship of taxons described in the work of of B.W. Skvortzow and C.I. Meyer “A contribution to the diatoms of Baikal Lake”. “Proceedings of the Sungaree River Biological Station”, 1928, Vol. 1, No. 5. Bulletin of the Moscow Society of Naturalists, Biological section 79(1): 152–156. [In Russian]

[B23] Skabichevsky AP (1977) Algae fouling Chaetomorphs of the sublittoral of the eastern Baikal shore. Prirodnye kompleksy nizshikh rastenii Zapadnoi Sibiri [Natural Complexes of Lower Plants in Western Siberia]. Nauka, Novosibirsk, Russia, 121–132. [In Russian]

[B24] Skabitschewsky AP (1952) Ad systematicam diatomarum baikalensium = Zur Systematik der Diatomeen des Baikal-Sees. Botanicheskie materialy otdela sporovykh rasteniy Botanicheskogo instituta imeni V.L. Komarova, Akademii Nauk S.S.S.R. 8: 36–45. [In Russian]

[B25] Skvortzov BV (1946) New and little known species of algae, flagellatae, phycomicetae from Asia, America, Africa, as well as from the Japan and Ceylon islands, described in 1931–45, with 18 tables of drawings (figures). Zapiski Kharbinskogo Obshchestva Estestvoispytatelei i Etnografov [Proceedings of the Harbin Society of Natural History and Ethnography] 2. Botany, 1–34. [In Russian]

[B26] Skvortzow BW (1937) Bottom diatoms from Olhon Gate of Lake Baikal. Philippine Journal of Science 62: 293–377.

[B27] Skvortzow BW Meyer CI (1928) A contribution to the diatoms of Baikal Lake. Proceedings of the Sungaree River Biological Station 1: 1–55.

[B28] Turland NJ Wiersema JH Barrie FR Greuter W Hawksworth D Herendeen PS Knapp S Kusber W-H Li D-Z Marhold K May TW McNeill J Monro AM Prado J Price MJ Smith GF [Eds] (2018) International Code of Nomenclature for algae, fungi, and plants (Shenzhen Code) adopted by the Nineteenth International Botanical Congress Shenzhen, China, July 2017. Koeltz Botanical Books, Glashütten. 10.12705/Code.2018 [accessed on 30 Aug 2023]

[B29] Werum M Lange-Bertalot H (2004) Diatoms in springs from Central Europe and elsewhere under the influence of hydrogeology and anthropogenic impacts. Iconographia Diatomologica 13: 1–480.

[B30] Williams DM Reid G (2001) A bibliography of the scientific work of Boris V. Skvortzov (1896–1980) with commentary on the publications concerning diatoms (Bacillariophyta). Bulletin of the British Museum Natural History. Bot. Ser. 31(2): 89–106.

[B31] Wojtal AZ (2009) The Diatoms of Kobylanka Stream near Kraków (Wyżyna Krakowsko-Częstochowska Upland, S. Poland). Polish Botanical Journal 54(2): 129–330.

[B32] Yurchak MI Gogorev RM Sokolova IV Kulikovskiy MS Glushchenko AM (2023) Nomenclature and taxonomy of two Baikal *Cocconeis* species (Bacillariophyta): *Cocconeisbaicalensis* and *Cocconeisskvortzowii*. Voprosy sovremennoi algologii (Issues of modern algology) 32(2): 22–24. 10.33624/2311-0147-2023-2(32)-22-25 [In Russian]

